# Longitudinal Associations Between Serum Cytokine Levels and Dementia

**DOI:** 10.3389/fpsyt.2018.00606

**Published:** 2018-11-19

**Authors:** Ju-Wan Kim, Robert Stewart, Hee-Ju Kang, Kyung-Yeol Bae, Sung-Wan Kim, Il-Seon Shin, Jin-Sang Yoon, Jae-Min Kim

**Affiliations:** ^1^Department of Psychiatry, and Depression Clinical Research Centre, Chonnam National University Medical School, Gwangju, South Korea; ^2^Institute of Psychiatry, King's College London, London, United Kingdom

**Keywords:** cytokines, dementia, geriatric psychiatry, inflammation, longitudinal studies

## Abstract

**Background:** The purpose of this study was to investigate whether long-term inflammation is related to the incidence of dementia in a prospective observational study.

**Methods:** In total, 732 Korean community-dwelling elderly people >65 years were evaluated at baseline. Of the 625 without dementia, 518 (83%) were followed over a 2.4-years period, and the incidence of dementia was determined. Cytokine [interleukin (IL)-1α, IL-1β, IL-6, IL-8, and tumor necrosis factor (TNF)-α] levels were measured at baseline and follow-up. The individual and combined effects of cytokine levels on dementia were evaluated after adjusting for potential covariates (lifestyle factors, demographics, disability, cognitive function, and presence of the APOE e4 allele) and a Bonferroni correction.

**Results:** Incident dementia was associated with increased serum cytokine levels after 2 years; the association remained significant for TNF-α, IL1-α, and IL-1β concentrations even after applying a Bonferroni correction. The analysis of the combined effects of the five cytokines showed independent associations between increases in the summed number of higher cytokine levels, between baseline and follow-up. However, incident dementia was not expected based on higher baseline pro-inflammatory cytokine levels.

**Conclusion:** Our results suggest that dementia may precede changes in serum cytokine levels and inflammatory processes, rather than resulting from elevated pro-inflammatory cytokines.

## Introduction

Dementia is a group of symptoms involving impairments in attention, memory, language, executive function, perception, and social cognition (1). As dementia has a high prevalence and a progressive, irreversible course in older adults (2, 3), it has become a global challenge for public health (4, 5). Understanding the etiology of dementia is essential for early diagnosis and treatment. Dementia has complex and heterogeneous etiologies, including amyloid plaques, tau-pathy, and cerebrovascular disease (6–8). Recently, the inflammatory process has received attention in the pathogenesis of dementia (9, 10).

Cytokines play a pivotal role in regulating the inflammatory response. The involvement of cytokines in dementia is supported by studies (11–13) showing that the levels of pro-inflammatory cytokines [e.g., tumor necrosis factor alpha (TNF-α), interleukin (IL)-1α, IL-1β, IL-6, and IL-8] are altered. A recent meta-analysis study reported significantly higher levels of the proinflammatory cytokines TNF-α, IL-6, IL-1β, IL-2, and IL-18 in peripheral blood samples of patients with Alzheimer's disease (AD) compared with a control group (14). However, causal relationships cannot be confirmed from case-control and cross-sectional investigations because increases in pro-inflammatory processes could be caused by disease pathophysiology (15, 16). Therefore, longitudinal studies are needed for clarification, although such studies have been scarce and have reported inconsistent findings. Some previous longitudinal studies have reported that the incidence of dementia is associated with TNF-α, IL-6, and C-reactive protein (CRP) concentrations (10, 17, 18), whereas other studies have found no relationship between baseline inflammation and future risk of dementia after adjusting for confounding factors (19, 20). These studies of inflammation-related dementia risk were conducted at a single time point, providing only the temporality of disease progression. Therefore, whether increased cytokine levels at a baseline evaluation can predict dementia risk remains unknown. To address these limitations, we analyzed data from a 2.4-years longitudinal cohort study to investigate prospectively the associations between five serum cytokine levels estimated at baseline and at follow-up with the incidence of dementia.

## Methods

### Study overview and participants

This prospective cohort study was carried out from 2001 to 2003 in Kwangju, South Korea. Details of the study design have been described previously (21). Briefly, 732 community dwelling subjects aged >65 years from Kwangju, South Korea, were recruited from national resident registration lists. Of the 732 participants, 625 did not receive a dementia diagnosis at the baseline evaluation. Among these, 518 (83%) finished all assessments at follow-up. A secondary analysis was carried out with these 518 participants. The study was performed in accordance with the Declaration of Helsinki. Written informed consent was obtained for the study, and this study was approved by the Chonnam National University Hospital Institutional Review Board.

### Baseline sampling and assessments

Examinations included a fully structured diagnostic interview for AD and vascular dementia, peripheral blood sampling for five serum pro-inflammatory cytokines, and a formal evaluation of covariate factors.

#### Dementia evaluation

Dementia assessments were performed at the baseline and follow-up. Examinations included the Mini-Mental State Examination (MMSE) (22), the Instrumental Activities of Daily Living Scale (23), the Clinical Dementia Rating scale (24), history, and a neurological examination. Clinical researchers assigned consensus diagnoses using standard criteria for dementia (1), AD (25), and vascular dementia (26). If AD and vascular dementia pathologies were mixed, they were diagnosed as either, using the criteria. In this study, we divided the participants into two different groups of incident dementia and no incident dementia.

#### Biochemical analyses

Blood samples were collected from the participants in a fasting state and were taken in the morning when possible. The blood samples were collected in EDTA tubes, centrifuged, separated into plasma aliquots, and frozen at −70°C within 2 h of collection. Biochemical assays were conducted for five serum cytokines: IL-1α, IL-1β, IL-6, IL-8, and TNF-α. Serum cytokine levels were analyzed using a solid-phase sandwich enzyme-linked immunosorbent assay (ELISA) kit (Invitrogen, Camarillo, CA, USA) according to the manufacturer's instructions. To reduce assay variation, all specimens were analyzed on the same day, in duplicate, in random order, by a technician blinded to the participant's status. The inter-assay coefficients of variation were 4–8% for IL-1α, 5–9% for IL-1β, 6–9% for IL-6, 3–6% for IL-8, and 5–7% for TNF-α. The intra-assay coefficients of variation were 4–6% for IL-1α, 4–7% for IL-1β, 4–5% for IL-6, 2–4% for IL-8, and 3–6% for TNF-α.

#### Covariates

Several potential factors that may be associated with serum cytokine levels were investigated based on previous studies (6–8). Age, gender, and education were recorded. Depression was evaluated by the community version of the Geriatric Mental State schedule (GMS) (27). Functional disability was measured by the Korean version of the World Health Organization Disability Assessment Schedule II (WHODAS II) (28). Smoking status was investigated and participants were categorized into current smokers or not current (ex- and never) smokers. History of alcohol use was ascertained from the participants, and verified by family members. Low risk alcohol drinking was defined, based on consuming more than 14 drinks per week during the previous 3 months for men or more than 7 drinks per week for women, following the guidelines from the National Institute of Alcohol Abuse and Alcoholism (29). Physical activity was checked at baseline by asking about work and leisure activities on a 4-point scale (not at all active, not very active, fairly active, and very active) according to the standard protocol (30). A summary vascular risk score was calculated by summing self-reported disorders (stroke, heart disease, hypertension, and diabetes), hypercholesterolemia (fasting cholesterol >200 mg/dl), and obesity (body mass index >25 kg/m^2^). The apolipoprotein E (APOE) genotype was reflected as a covariate in analyses of the relationship with AD.

### Follow-up assessments

The follow-up was carried out in 2003 (31). Attempts were made to follow up 2 years after the baseline appointment (mean interval [standard deviation (SD)], 2.4 (0.3) years). Blood samples were collected to determine five serum pro-inflammatory cytokines, centrifuged within the hour, and stored at −70°C. The same biomedical assays were carried out using the ELISA method described above. Clinical researchers assigned consensus diagnoses using standard criteria (1, 25, 26).

### Statistical analysis

**Figure 1:**
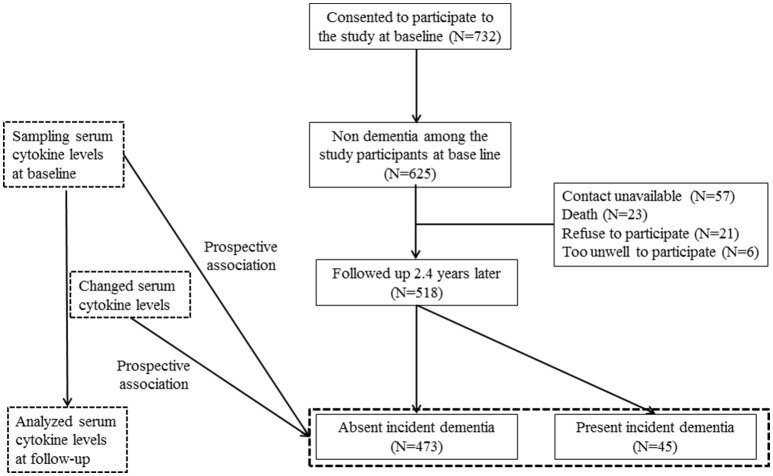
Flow chart of the study.

The study design is outlined in Figure 1. Associations between baseline characteristics, demographics, assessment scales (MMSE, WHODAS II, and GMS depression) (27, 28, 32), smoking, alcohol, physical activity, and vascular risk score with incident dementia were analyzed using t- or χ^2^-tests as appropriate. Results with significant associations (*p*-value < 0.05) were reflected in subsequent multivariate analyses. Unadjusted associations between the incidence of dementia and baseline serum cytokine concentrations and changes (from baseline to follow-up) were investigated using the Mann–Whitney *U*-test. The baseline levels and changes in levels were recorded as binary values of “lower” or “higher” (i.e., below or above the median value). Odds ratios and 95% confidence intervals were calculated for the associations between the baseline cytokine values and increases in cytokine levels during the follow-up in subjects with dementia. These associations were analyzed before and after adjusting for significant baseline covariates using logistic regression models. Because cytokines have demonstrated additive and synergistic effects on AD (21), the combined effects of the pro-inflammatory cytokines were calculated by summing the number of cytokines with higher levels at baseline and the number of cytokines with higher levels at the follow-up, and then assigning ordinal values (0, 1, 2, 3, 4, 5) to each. Associations between baseline values and the summed number of higher cytokine levels with the incidence of dementia were assessed initially by the χ^2^-test for a linear trend (i.e., 1 df), and then with the same logistic regression models to adjust for covariates. The Bonferroni correction was applied to maintain an overall type I error rate of 0.05 against the multiple comparisons. All statistical analyses were performed with SPSS software (ver. 21.0; SPSS Inc., Chicago, IL, USA).

## Results

### Participant characteristics

The recruitment procedure for the baseline and follow-up aspects of the study and distribution of dementia diagnoses are shown in Figure 1. Among the 625 subjects without dementia at the baseline evaluation, 518 (83%) completed all evaluations. At follow-up, incident dementia [mean interval (SD), 2.4 (0.3) years] was diagnosed in 45 participants (8.7%): 34 (6.6%) had AD, seven (1.4%) had vascular dementia, and four had “other” dementia (0.8%). Median [interquartile range (IQR)] IL-1α, IL-1β, IL-6, IL-8, and TNF-α levels were 40.3 (32.3–48.3), 12.0 (11.1–12.9), 9.5 (8.9–10.5), 20.1 (18.7–23.1), and 40.7 (37.5–43.9) pg/ml, respectively. Baseline characteristics are compared by the dementia diagnosis in Supplementary Table 1. Incident dementia was associated with older age, lower education, lower MMSE score, higher WHODAS II score, lower physical activity, and presence of the APOE e4 allele. The median (IQR) changes from baseline to follow-up for IL-1α, IL-1β, IL-6, IL-8, and TNF-α levels were +0.3 (0.3–1.9), +0.6 (0.4–0.8), +0.6 (0.6–4.0), +0.3 (0.1–1.8), and +1.2 (1.0–3.9), pg/ml, respectively. Baseline levels of and follow-up changes in serum cytokine concentrations according to the dementia diagnosis are shown in Supplementary Table 2.

### Baseline and follow-up concentrations of serum cytokines by dementia incidence

Unadjusted associations between serial serum cytokine levels and incident dementia are summarized in Table 1. Incident dementia was associated with increases in all five serum cytokines during the follow-up, and the strength of the significance remained for TNF-α, IL1-α, and IL-1β after applying the Bonferroni correction. Incident dementia was associated with a higher IL-8 level at baseline, and the strength of the significance remained after applying the Bonferroni correction. The same analyses were repeated by dementia diagnosis and are presented in Supplementary Table 2. Incident AD was significantly associated with increases in IL1-α, IL-1β, and IL-6 levels but not with baseline cytokine levels after applying Bonferroni corrections. Incident vascular dementia was not associated any cytokine value.

**Table 1:** Baseline levels of and follow-up changes in serum cytokine concentrations by incident dementia status.

	**No incident dementia (*N* = 473)**	**Incident dementia (*N* = 45)**	*P*-value^*^
**BASELINE VALUES**			
Tumor necrosis factor-α	40.3 (32.5–48.5)	40.7 (31.7–50.4)	0.921
Interleukin-1α	12.1 (11.1–12.9)	11.9 (11.2–14.1)	0.505
Interleukin-1β	9.5 (8.9–10.5)	9.3 (8.8–10.5)	0.981
Interleukin-6	20.0 (18.0–22.3)	21.0 (17.7–25.0)	0.146
Interleukin-8	40.4 (37.3–43.5)	43.0 (40.5–50.3)	**<0.001**
**FOLLOW-UP VALUES**			
Tumor necrosis factor-α	42.1 (36.5–47.7)	42.1 (37.1–47.1)	0.469
Interleukin-1α	13.2 (12.2–14.2)	13.9 (13.2–14.6)	**0.001**
Interleukin-1β	10.1 (9.5–11.1)	11.2 (10.0–12.6)	**<0.001**
Interleukin-6	22.1 (19.5–24.7)	24.6 (22.6–28.1)	**<0.001**
Interleukin-8	41.1 (38.4–44.6)	45.0 (38.5–53.5)	**0.002**
**CHANGED VALUES**			
Tumor necrosis factor-α	+1.2 (1.0–1.4)	+4.5 (−0.3–6.0)	**0.002**
Interleukin-1α	+0.3 (0.3–1.9)	+1.8 (1.1–2.0)	**0.001**
Interleukin-1β	+0.6 (0.6–0.7)	+2.2 (0.0–2.6)	**<0.001**
Interleukin-6	+0.6 (0.6–3.3)	+4.8 (0.8–6.0)	0.005
Interleukin-8	+0.3 (0.0–0.6)	+3.6 (−3.9–7.6)	0.100

* *Mann Whitney test, Bold character denotes statistical significance after Bonferroni correction*.

*Data are median (IQR)*.

### Binary higher baseline levels and increasing follow-up level categories of serum cytokines by incident dementia status

The logistic regression results show a positive relationship between baseline and follow-up cytokine levels with the incidence of dementia (Table 2). Incident dementia was associated with IL-8 levels at baseline in unadjusted analyses. However, the association was not significant after adjusting for covariates. Incident dementia was significantly associated with higher levels of all five cytokines before and after adjustment, even after applying the Bonferroni correction. Incident dementia was not associated with higher pro-inflammatory cytokine level category at baseline before or after adjusting for covariates.

**Table 2:** Associations of incident dementia with (binary) higher baseline cytokine levels and (binary) more pronounced increase in levels over follow-up.

	**Unadjusted**	**Adjusted^a^**
**BASELINE HIGHER LEVELS**		
Tumor necrosis factor-α	1.05 (0.57–1.93)	0.83 (0.43–1.60)
Interleukin-1α	0.97 (0.47–1.59)	1.29 (0.65–2.56)
Interleukin-1β	0.78 (0.42–1.45)	1.09 (0.56–2.14)
Interleukin-6	1.16 (0.63–2.14)	1.22 (0.63–2.36)
Interleukin-8	2.67 (1.37–5.21)^†^	2.56 (1.25–5.23)^*^
**MORE PRONOUNCED INCREASE IN LEVELS**		
Tumor necrosis factor-α	**7.83 (3.92–15.63)**^‡^	**8.44 (4.02–17.70)**^‡^
Interleukin-1α	**7.26 (3.31–15.95)**^‡^	**8.20 (3.58–18.82)**^‡^
Interleukin-1β	**7.16 (3.64–14.09)**^‡^	**7.19 (3.47–14.94)**^‡^
Interleukin-6	**5.23 (2.63–10.40)**^‡^	**5.01 (2.43–10.32)**^‡^
Interleukin-8	**3.80 (2.02–7.16)**^‡^	**3.73 (1.90–7.32)**^‡^

a *Adjusted for age, education, scores on Mini-Mental State Examination and World Health Organization Disability Assessment Scale II, APOE and physical activity*.

* p-value < 0.05;

† p-value < 0.01;

‡ p-value < 0.001.

Bold character denotes statistical significance after Bonferroni correction.

*Data are displayed as odds ratios (95% confidence intervals)*.

### Sums of higher baseline cytokine levels and increases in cytokine levels by dementia incidence

**Figure 2:**
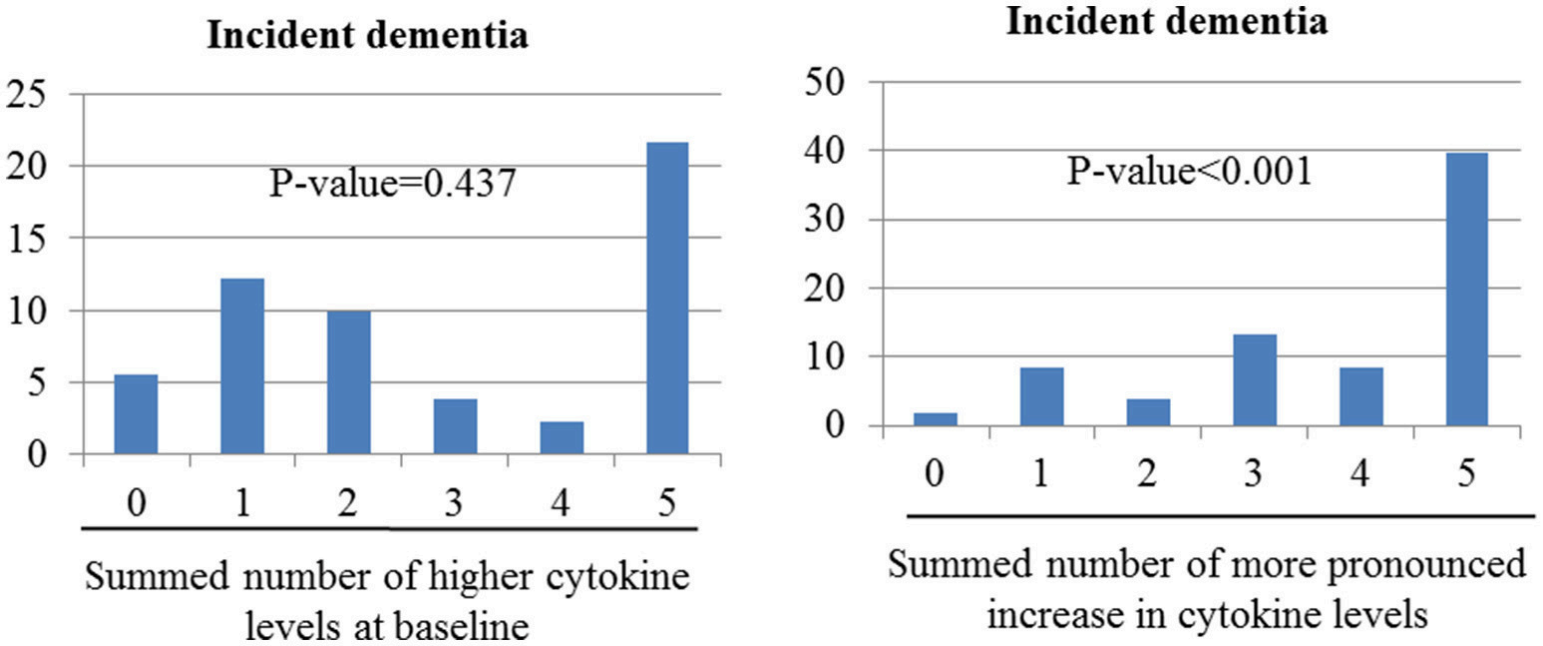
Associations of incident dementia with number of cytokine levels categorize as high at baseline or as showing more pronounced increase over the follow-up period. Odds ratios (95% confidence intervals) for the association between incident depression and increased summed number of higher cytokine levels at baseline was 1.08 (0.88–1.33), *p*-value = 0.437; and between incident dementia and increased summed number of changed higher cytokine levels was 1.91 (1.55–2.34), *p*-value < 0.001 after adjustment for sex, cognitive function, disability, physical activity, and presence of the APOE e4 allele.

Figure 2 shows the combined effects of baseline and follow-up cytokine levels on dementia incidence. Incident dementia was associated with increases in the number of changes to higher cytokine levels during follow-up in unadjusted analyses (χ^2^ = 50.91; *p*-value < 0.001). However, no association was found between the incidence of dementia and increases in the summed number of higher cytokine levels at baseline (χ^2^ = 0.604; *p* = 0.438). After adjusting for older age, lower education, lower MMSE score, higher WHODAS II score, lower physical activity, and presence of the APOE e4 allele, the incidence of dementia increased significantly with increases in the summed number of changes to higher cytokine levels at follow-up. No association was found between incident dementia and increases in the summed number of higher cytokine levels at baseline.

## Discussion

The principal findings of this study were that the incidence of dementia was significantly associated with increases in all five serum cytokines, considered as both continuous and dichotomized variables, at follow-up, and the strength of these associations remained significant after applying the Bonferroni correction and relevant adjustments. The analysis of the combined effects of the five cytokines showed independent associations between increases in the summed number of higher cytokine levels between baseline and follow-up before and after adjustment. The incidence of dementia was only predicted by higher IL-8 levels at baseline, but this association was no longer significant in a multivariate analysis after applying the Bonferroni correction.

Inflammation may be an important mechanism underlying dementia (33, 34); however, it is unclear whether the inflammatory response is associated with the onset of dementia or is an outcome of dementia. To understand how inflammatory cytokines change, and possibly exert negative effects in dementia, a prospective cohort study with numerous inflammatory markers is needed. Very few studies have incorporated data from multiple time points in a clinical sample (35–37). Among them, only one study reported no association between AD and longitudinal inflammation (35), and two studies described an association between increases in inflammatory markers from baseline to follow-up and subsequent AD (36, 37). In this study, incident dementia was significantly associated with increases in cytokine levels during the study period. Several potential mechanisms might explain these findings. First, the level of inflammation may serve as a marker of active pathological processes. Hyperphosphorylation of the tau protein forming neurofibrillary tangles and amyloid beta (Aβ) accumulation in plaques are trademarks of AD progression (38). The abnormal Aβ clearance process results from the distortion of astrocytes or microglia to a pro-inflammatory state. This process is represented by elevated levels of pro-inflammatory cytokines and dysregulation of clear Aβ, and it leads to Aβ accumulation and worsened immune activation (39). In this study, 41 of 45 (91.1%) participants with dementia had AD and vascular dementia. Although the etiologies of AD and vascular dementia may be different, and thus lead to different disease outcomes, they may begin as a similar cascade of cytokine production in response to neuronal injury (8, 38, 39). Increased cytokine levels were significantly associated with incident AD but not with vascular dementia. However, due to the small sample size, there is a possibility of a type II error in the non-significant associations with vascular dementia. Second, it is also possible that cytokines increased due to causes other than dementia during the follow-up period. For example, normal aging can cause an increase in peripheral cytokines (33, 40). Our longitudinal observations support this hypothesis. Five cytokines increased during the follow-up period in both the dementia and control groups. The term sensitization is often used to describe induction of an immune response. Here, the incidence of dementia was associated with an increase in cytokine levels, but not with the absolute concentrations of baseline cytokine levels. Therefore, when conditions other than aging occur that can accelerate inflammation, such as amyloid plaques and neurofibrillary tangles (9), depression (9), genetic conditions (41), infections (42), trauma, and vascular events (8), immune sensitization can occur, which can accelerate cognitive impairment.

Previous studies of cross-sectional cytokine levels as predictors of the incidence of dementia have resulted in heterogeneous findings (35, 43–45). Those studies were limited by differences in study design, such as participant characteristics. In our study, baseline IL-8 level was associated with incident dementia in unadjusted analyses, but was not significant in the multivariate analysis after applying the Bonferroni correction. Furthermore, the impacts of cytokines have been revealed to be synergistic and, given the inconsistent outcome of a single cytokine level, the combined effect of multiple cytokines represents a more practical approach (21, 46). No associations were detected when high cytokine levels were summed. Together, these data do not support the hypothesis that cytokines result in dementia-related dysfunction but, instead, suggest that cytokine levels are elevated by dementia-related processes.

This study has several strengths. Cytokine levels were assessed serially, which could help to clarify causal relationships. In addition, the follow-up rate was reasonable and a number of potential covariates were considered in the analyses. This study also has several limitations. First, due to limited resources, we only measured five cytokines, and other important anti-inflammatory (e.g., IL-4 and IL-10) and pro-inflammatory (e.g., IL-13 and IL-18) cytokines were not evaluated (47). It is important to determine the exact function of various cytokines and disease progression. Overproduction of several cytokines could result in damage to neurons, and these cytokines may play a role in the progression of dementia, but their protective effects have also been considered (47). Second, we collected blood samples from the periphery to measure inflammation in the central nervous system. Several studies have been conducted using imaging technology, including positron emission tomography, to measure neuroinflammation directly in subjects with dementia (48, 49). More studies are required to demonstrate the relationships between these cytokines and dementia. Third, due to protein degradation, it is recommended that blood samples be examined immediately. In this instance, due to technical difficulties, they were centrifuged at 3,000 rpm for 10 min, and sera were stored at −70°C until we finished specimen collection. However, most studies follow this procedure (20, 50). Fourth, the small set of covariate-adjusted analyses leaves the possibility of confounding bias. For example, we adjusted for age, education, MMSE, WHODAS, and physical activity but not for alcohol, smoking, or body mass index, which did affect the associations between cytokine levels and dementia, although these factors were not significantly associated with incident dementia at the follow-up (Supplementary Table 1). In particular, we could not provide an accurate diagnosis for smoking and alcohol use. Varying the cutoff points may lead to a different interpretation of the results of a trial. Thus, our adjustment might have been suboptimal. Fifth, conclusions regarding dementia that fully reflect the complex disease status are improbable, as stated above. In this study, due to a limitation in the study design, the probability that increases in cytokines may arise transiently, as in acute infectious disease, or continuously because of chronic inflammatory disease, may have influenced the heterogeneity, and both diseases may contribute to incident dementia. In addition, it was difficult to diagnose mixed dementia due to a lack of brain imaging data. However, this limitation is common in most epidemiological studies. Finally, because this study did not specify the timing of the dementia diagnosis, it was not possible to state clearly that dementia preceded inflammation. Furthermore, only 2 years of follow-up visits were carried out. Because dementia progresses over decades, having a longer follow-up is crucial to identifying the developmental trajectory of the disease. Additional follow-up studies will be needed to address these limitations.

Our findings suggest that incident dementia may lead changes in serum cytokine levels and inflammation, rather than resulting from elevated pro-inflammatory cytokines. It has been reported that patients with dementia, particularly those at more advanced stages, are at a higher risk for developing stroke and depression (51, 52), which could, theoretically, be mediated by inflammation. As mentioned above, our findings must be replicated in future studies with more cytokines. Furthermore, interactions between cytokine levels and genes need further assessment, as there is sufficient evidence that the transcriptional activity of specific polymorphisms influences cytokine production (53).

## Author contributions

J-WK conducted the data analysis and drafted the article. RS and H-JK helped to analyze the data and to draft the article. K-YB, S-WK, I-SS, and J-SY helped to recruit the participant and perform dementia assessment and management. All authors approved the final version of manuscript to be published. J-MK had full access to all of the data in the study and take responsibility for the integrity of the data and the accuracy of the data analysis.

### Conflict of interest statement

The authors declare that the research was conducted in the absence of any commercial or financial relationships that could be construed as a potential conflict of interest.
