# Supplementary material for: Longitudinal Associations Between Serum Cytokine Levels and Dementia
Source: Front Psychiatry. 2018 Nov 19;9:606. doi: 10.3389/fpsyt.2018.00606 (PMC6252389; doi:10.3389/fpsyt.2018.00606)
Supplement: Supplementary file 1 [file Table_1.DOCX]

| **Supplementary Table 1** Baseline characteristics by incident dementia status | | | |
| --- | --- | --- | --- |
|  | No incident dementia  (N=473) | Incident dementia  (N=45) | P-value^*^ |
| Demographic characteristics |  |  |  |
| Age, mean (SD) years | 71.6 (4.9) | 74.3 (5.7) | 0.001 |
| Female sex, N (%) | 254 (53.7) | 28 (62.2) | 0.273 |
| Education, median (IQR) years | 3 (0-6) | 0 (0-2) | <0.001 |
| Assessment scales |  |  |  |
| MMSE, mean (SD) scores | 23.7 (4.4) | 20.5 (4.2) | <0.001 |
| WHODAS II, median (IQR) scores | 2.2(0-6) | 5.4(1-12) | 0.001 |
| GMS depression, N (%) | 50 (10.6) | 7 (15.6) | 0.307 |
| Lifestyle characteristics |  |  |  |
| Current smoker, N (%) | 199 (42.1) | 16 (35.6) | 0.397 |
| High alcohol intake, N (%) | 141 (29.8) | 14 (31.1) | 0.855 |
| Low physical activity, N (%) | 118 (24.9) | 24 (53.3) | <0.001 |
| Vascular risk score, median (IQR) | 1 (0-2) | 1 (1-2) | 0.121 |
| Body mass index, mean (SD) kg/m^2^ | 22.9 (3.4) | 22.2 (3.5) | 0.245 |
| APOE e4 allele, N (%) | 74 (15.6) | 14 (31.1) | 0.008 |

MMSE: Mini-Mental State Examination; WHODAS II: World Health Organization Disability Assessment Scale II; GMS: Geriatric Mental State schedule

^*^t-test or χ^2^ test as appropriate.
