# Supplementary material for: Longitudinal Associations Between Serum Cytokine Levels and Dementia
Source: Front Psychiatry. 2018 Nov 19;9:606. doi: 10.3389/fpsyt.2018.00606 (PMC6252389; doi:10.3389/fpsyt.2018.00606)
Supplement: Supplementary file 2 [file Table_2.docx]

| **Supplementary Table 2.** Baseline levels of and follow-up changes in serum cytokine concentrations by dementia diagnosis. Data are median (IQR). | | | | | | | |
| --- | --- | --- | --- | --- | --- | --- | --- |
|  | No dementia  (N=473) | Incident AD  (N=34) | Incident Vascular dementia (N=7) | P-values^a^ | | | P-values^b^ |
| **Baseline values** |  |  |  | |  |  | |
| Tumor necrosis factor- α | 40.3 (32.5-48.5) | 39.6 (30.8-47.8) | 44.1 (33.8-59.6) | | 0.583 | 0.298 | |
| Interleukin-1α | 12.1 (11.1-12.9) | 11.7 (11.7-13.3) | 14.3 (12.1-20.9) | | 0.452 | 0.082 | |
| Interleukin-1β | 9.5 (8.9-10.5) | 9.2 (8.4.-10.0) | 9.3 (9.3-11.7) | | 0.284 | 0.357 | |
| Interleukin-6 | 20.0 (18.0-22.3) | 19.5 (18.8-25.4) | 24.0 (21.3-34.8) | | 0.930 | 0.017 | |
| Interleukin-8 | 40.4 (37.3-43.5) | 47.0 (43.2-51.6) | 53.5 (42.2-80.0) | | 0.005 | 0.157 | |
| **Changed values** |  |  |  | |  |  | |
| Tumor necrosis factor- α | +1.2 (1.0-1.4) | 4.5 (-3.8-8-8) | 4.5 (0.7-4.5) | | 0.014 | 0.328 | |
| Interleukin-1α | +0.3 (0.3-1.9) | 1.8 (1.2-2.3) | 1.8 (-3.7-1.8) | | **<0.001** | 0.773 | |
| Interleukin-1β | +0.6 (0.6-0.7) | 2.1 (0.6.-3.1) | 1.9 (-1.1-3.2) | | **<0.001** | 0.490 | |
| Interleukin-6 | +0.6 (0.6-3.3) | 4.6 (0.8-5.6) | 2.4 (-9.4-7.4) | | **0.003** | 0.854 | |
| Interleukin-8 | +0.3 (0.0-0.6) | 2.9 (-2.8-7.6) | 7.2 (-13.1-7.2) | | 0.073 | 0.667 | |

^a^p-value for no dementia versus incident AD using Mann whitney test. ^b^p-value no dementia versus Incident Vascular dementia using Mann whitney test.

Bold character denotes statistical significance after Bonferroni correction
